# Evolutionarily stable conjectures and other regarding preferences in duopoly games

**DOI:** 10.1007/s00191-017-0529-1

**Published:** 2017-08-26

**Authors:** Ilkka Leppänen

**Affiliations:** School of Business and Economics, Loughborough University, Loughborough, LE11 3TU UK

**Keywords:** Duopoly, Conjectural variations, Other regarding preferences, Evolutionary stability, D43, B52, C73

## Abstract

We study the evolutionary selection of conjectures in duopoly games when players have other regarding preferences, i.e. preferences over payoff distributions. In both the Cournot and Bertrand duopoly games, the consistent conjectures are independent of other regarding preferences. Both duopoly games have evolutionarily stable conjectures that depend on other regarding preferences but that do not coincide with the consistent conjectures. For increasingly spiteful preferences, the evolutionarily stable conjectures implicate low quantities in the Cournot game and high prices in the Bertrand game, whereas the inverse relationships hold for the consistent conjectures. We discuss our findings in the context of ultimate and proximate causation.

## Introduction

Could it be the case that conjectures are consistent but that subjects are maximizing something other than profit? – Charles (Holt 1985)

In many games players maximize something other than profit. In the strategic delegation literature a firm’s owners incentivize managers to include market share or the relative profits with respect to rivals into their objectives (Vickers 1985; Fershtman et al. 1991). In mixed oligopoly models, a welfare-maximizing public firm competes with profit-maximizing private firms (De Fraja and Delbono 1989). The motive for maximizing other things than profit can also be non-strategic. A large body of literature in experimental economics has demonstrated that players have other regarding preferences (ORPs) that can be included into the maximization problem by complementing the utility functions with such factors as concerns for efficiency or reciprocity (Cooper and Kagel 2016).

Various models of strategic behavior also take beliefs into account. A traditional way to model beliefs in the industrial organization literature is to assume that firms conjecture that competitors react to variations in own strategies by nonzero variations in their strategies. As in the case of ORP models, the conjectural variations models can represent many different outcomes in a single framework (Varian 1992). However, conjectural variations have been criticized as imposing a dynamic process of belief formation into a static framework (e.g. Makowski 1987). Presently, conjectural variations are modeled in contexts that have some sort of an implicitly or explicitly dynamic nature (Figuières et al. 2004). In these models, the conjectural variations equilibria are interpreted as shortcuts to modeling outcomes of dynamic games (Dockner 1991; Cabral 1995). Alternatively the conjectures can be assumed to result from behavior that is boundedly rational, either in belief formation (Friedman and Mezzetti 2002; Jean-Marie and Tidball 2006) or in short-term fitness maximization in the presence of evolutionary selection pressure on the conjectures (Dixon and Somma 2003; Müller and Normann 2005; Possajennikov 2009). Common to these bounded rationality approaches is that they justify the consistent conjecture, i.e. a conjecture that represents correctly anticipating the competitor’s reaction (Bresnahan 1981).

The appeal of the evolutionary approach is that it can also justify the origin of the conjectures. In the models studying the evolutionary selection of conjectures in infinite populations, the evolutionarily stable conjecture coincides with the consistent conjecture (Müller and Normann 2005). Possajennikov (2009) argues that this occurs because the first-order conditions of payoff maximization and fitness maximization coincide and the players solve the correct maximization problem.

Our motivation in this paper is to demonstrate cases when the consistent conjecture is *not* justified by evolutionary arguments. We use the indirect evolutionary approach to derive conjectures in a model where other-regarding (altruistic or spiteful) behavior is possible. We show that the evolutionarily stable conjecture does not coincide with the consistent conjecture if the players maximize a simple utility function that takes ORPs into account, thus contrasting with the results obtained by Müller and Normann (2005). We consider the two classical models of duopoly competition, quantity competition (Cournot) and price competition (Bertrand). Both games have evolutionarily stable conjectures with ORPs that do not coincide with the consistent conjectures.

The model of ORPs that we consider is a linear rule that imposes a concern for the total payoff distribution. Varying the ORP parameter allows the representation of altruism, spite, or self-regard. A concern for relative payoffs can be modeled with a change of the parameter. As this model has only a single parameter, it is selected over competing and more complex models of other-regarding behavior (such as the inequity aversion model of Fehr and Schmidt (1999) or the egocentric altruism model of Cox et al. 2007) for its analytical tractability. Although the ORP parameter is symmetric among the players, the model captures the essence of distributional preferences.

Some evolutionary models in the literature subject the ORP parameter to selection pressure and derive conditions for stability of altruism or spite (Bester and Güth 1998; Possajennikov 2000). In our model only the conjecture is shaped by selection pressure but the ORP parameter is free of that pressure. Thus, in an evolutionary sense the phenotypic variability is maintained in the model via the ORP parameter. We find that this variability is maintained also in the conjectures themselves, i.e. the conjectures (beliefs) depend directly on ORPs.

We analyze the Cournot duopoly game in Section 2 and the Bertrand duopoly game in Section 3. In each of these sections we first derive the consistent conjecture and then analyze the evolutionary stability. Section 4 discusses the results and Section 5 contains all the proofs.

## The Cournot duopoly game

### The model

There are two players in the model. In the rest of this article the player is indexed by *i* and the other player is referred to as *j*, such that *i*≠*j*. The players make simultaneous decisions in a one-shot game. They choose quantities $x_{i} \in \mathbb {R}^{+}\cup \{0\}$ as strategic variables. The inverse demand functions are *a* − *x*_*i*_ − *b* *x*_*j*_, where $a\in \mathbb {R}^{+}$ and $b\in (0,1)\subset \mathbb {R}$. We restrict the product differentiation parameter *b* to be strictly less than one for the existence of consistent conjectures (Bresnahan 1981). We also assume that *a* is sufficiently large with respect to the market total quantity so that the inverse demand function is positive for all reasonable-sized quantities. The cost function for player *i* is $c{x_{i}^{2}}$, where $c\in \mathbb {R}^{+}\cup \{0\}$. The payoff function for player *i* is linear-quadratic and given by

1 $$ f_{i} (x_{i},x_{j}) = x_{i} (a-x_{i}-bx_{j}) - c{x_{i}^{2}}. $$

The market has complete information, so the payoffs are known to both players. Both players experience utility that depends linearly on both players’ market payoffs. The utility function for player *i* is given by

2 $$ u_{i} (x_{i},x_{j}) = f_{i}(x_{i},x_{j}) + t f_{j} (x_{i},x_{j}). $$

The ORP parameter *t* assumes values on the open interval $(-1,1)\subset \mathbb {R}$. When *t* > 0 the player is altruistic, when *t* < 0 he is spiteful, and when *t* = 0 the preferences are self-regarding. We assume that the value of *t* is a fixed characteristic of a player and, importantly, is not subject to the players’ discretion. For simplicity, we also assume that *t* is equal for both players and this is common knowledge. The plausibility of this assumption is assessed at Section 4.

The conjectures are beliefs of player *i* about how player *j* reacts to a variation in own quantity *x*_*i*_. In other words, a unit change in own quantity results in a non-unit change in the total quantity. As is conventional in the conjectural variations literature, we assume that the conjectures are constant. Therefore, a player does not form a belief about the whole reaction function but only about its slope at specific values of the strategic variables. Player *i*’s conjecture is defined as

$$\frac{d x_{j}}{d x_{i}} (x_{i},x_{j})=r_{i}, $$

where we make the standard assumption that $r_{i} \in [-1,1]\subset \mathbb {R}$. This assumption captures the relevant results that range from perfect competition (*r*_*i*_ = −1) to collusion (*r*_*i*_ = 1). The conjectures can be made to appear in the first-order conditions of utility maximization obtained by total differentiation. Similar to Possajennikov (2009), we denote the left-hand side of player *i*’s first-order condition by defining a function

3 $$\begin{array}{@{}rcl@{}} F_{i}(x_{i},x_{j};r_{i}) :&=& \frac{\partial u_{i}}{\partial x_{i}}(x_{i},x_{j}) + r_{i}\frac{\partial u_{i}}{\partial x_{j}}(x_{i},x_{j}) \\ &=&a \,+\, a r_{i} t - 2 (1 \,+\, c) x_{i} \,-\, 2 (1 \!+ c) r_{i} t x_{j} - b (1 + t) (r_{i} x_{i} + x_{j}). \end{array} $$

The first-order condition *F*_*i*_(*x*_*i*_,*x*_*j*_;*r*_*i*_) = 0 defines implicitly the reaction functions $x_{i}^{*}(x_{j};r_{i})$. Because the payoff functions are quadratic, the reaction functions are linear. For the special case of *r*_*i*_,*r*_*j*_ = 0, the players have Nash conjectures and the corresponding equilibrium is the Cournot-Nash equilibrium (CNE).

#### **Lemma 1**

*The equilibrium quantities as function of arbitrary conjectures,* $x_{i}^{*}(r_{i},r_{j})$ *,* *exist for all feasible* *a*, *b*, *c* *and t* *and are given by*

4 $$ \begin{array}{lllllll} &x_{i}^{*}(r_{i},r_{j})=\\&\frac{a \left( b (1 + t) \left( -1 + r_{j} + (-1 + r_{i}) r_{j} t\right) - 2 (1 + c) (-1 + r_{i} r_{j} t^{2})\right)}{b^{2} (-1 + r_{i} r_{j}) (1 + t)^{2} - 2 b (1 + c) (r_{i} + r_{j}) (-1 + t^{2}) - 4 (1 + c)^{2} (-1 + r_{i} r_{j} t^{2})}. \end{array} $$

The conjectures are required to be constant in the strategic variables but they may depend on the other parameters *a*, *b*, *c* and *t*. To allow generality, we are only interested in symmetric conjectures *r*_*i*_ = *r*_*j*_ = *r* that are continuous in the full ranges of the parameters. The symmetry assumption of the conjectures is not restrictive because the payoff and utility functions are symmetric as well. By the chain rule, because the payoff function (1) is smooth, the utility function (2) is also smooth in the strategic variables.

#### *Remark 1*

By inspecting the derivative of the reaction function $x_{i}^{*}(x_{j};r)$ w.r.t *x*_*j*_ we can obtain information about its slope for different ranges of t and r. For zero ORPs, *t* = 0, the slope is − *b*/(2 + 2*c* + *b* *r*) < 0 and the reaction function is downward-sloping for any *r* ∈ [−1,1] in the Cournot duopoly game. For nonzero ORPs, *t* ≠ 0, the slope is −(2(1 + *c*)*r* *t* + *b*(1 + *t*))/ (2 + 2*c* + *b* *r*(1 + *t*)), and the reaction function is upward-sloping when

$$-1<r<-\frac{b}{1+c}, \quad -\frac{b}{b + 2 (1 + c) r}<t<1 $$

or when

$$0<r<1, \quad -1<t< -\frac{b}{b + 2 (1 + c) r}. $$

In other words, the downward-sloping reaction function is the standard case except for certain ranges of negative conjectures and altruistic preferences or for positive conjectures and spiteful preferences, in which cases the reaction function can slope upwards. Knowing the reaction function slopes helps us understand how equilibrium profits behave. Upwards-sloping reaction functions imply that quantities are complements and then increasing quantities increase profits as well. In the self-regarding case, the reaction functions always slope downwards and quantities are substitutes, but our analysis above indicates that quantities can become complements for nonzero ORPs and nonzero conjectures.

### Consistent conjectures

A consistent conjecture equals the actual reaction function slope at the equilibrium, i.e. at $x_{i}=x_{i}^{*}$. A consistent conjecture equilibrium (CCE) is a pair of quantities $(x_{i}^{*},x_{j}^{*})$ and a pair of symmetric conjectures (*r* ^∗^,*r* ^∗^) that solve

5 $$ r = -\frac{\partial F_{j} / \partial x_{i}(x_{j}^{*},x_{i}^{*};r)}{\partial F_{j} / \partial x_{j}(x_{j}^{*},x_{i}^{*};r)}. $$

Equation (5) gives a pair of equations that are obtained by applying the implicit function theorem on the first-order conditions *F*_*i*_(*x*_*i*_,*x*_*j*_,*r*) = 0 (Possajennikov 2009). In the Cournot duopoly game, this equation is for both players equal to

6 $$ r = -\frac{b + \left( b + 2 (1 + c) r\right) t}{2 + 2 c + b r (1 + t)}. $$

#### **Proposition 1**

*In Cournot competition with ORPs, the consistent conjecture is*

7 $$ r^{*} = -\frac{1+c-\sqrt{-b^{2}+(1+c)^{2}}}{b}. $$

#### *Remark 2*

Proposition 1 shows that the consistent conjecture does not depend on t in the Cournot duopoly game. Assuming ORPs, therefore, does not change the predictions of the consistent conjectures theory. This was already shown by Holt (1985), but we have generalized his result for nonconstant marginal cost and differentiated products. The consistent conjecture (7) is strictly negative for all feasible values of b and c, as is typical for Cournot competition (Bresnahan 1981).

### Evolutionarily stable conjectures

Evolutionarily stable (ES) conjectures are formed by selection pressure. Players with different conjectures are matched in duopoly pairs and those players who have the highest fitness pass their conjectures to future generations of players. In practice this can happen, e.g., through cultural evolution or when managers switch between firms. Alternatively, the conjectures may evolve through individual evolutionary learning (Arifovic and Maschek 2006) where beliefs (i.e. the conjectures) are updated as if they undergo natural selection. In this section, we assume that the population from which the conjectures are selected is infinite.^1^ The conjectures thus derived conform to the concept of evolutionarily stable (ES) strategies, introduced by Maynard Smith (1982). A strategy is evolutionarily stable if it cannot be invaded by any alternative mutant strategy.

The fitness of each player is determined by its payoff function (Bester and Güth 1998; Possajennikov 2000). The payoff functions are reformulated as fitness functions where quantities are given by equilibrium quantities and the relevant choice variables are the conjectures *r*_*i*_,*r*_*j*_. The ES conjectures can be solved from the fitness maximization problem (Possajennikov 2009)

$$\max_{r_{i}} f_{i} \left( x_{i}^{*}(r_{i},r_{j}),x_{j}^{*}(r_{i},r_{j})\right) $$

where $x_{i}^{*}(r_{i},r_{j})$ and $x_{j}^{*}(r_{i},r_{j})$ are the equilibrium strategies as functions of arbitrary conjectures. We know that the fitness function *f*_*i*_ is well-defined in *r*_*i*_,*r*_*j*_ because the equilibrium strategies $x_{i}^{*}(r_{i},r_{j}),x_{j}^{*}(r_{i},r_{j})$ exist for each feasible pair of conjectures *r*_*i*_,*r*_*j*_ (Lemma 1). Player *i*’s first-order condition for fitness maximization is

8 $$ \frac{\partial f_{i}}{\partial r_{i}} (x_{i}^{*}(r_{i},r_{j}),x_{j}^{*}(r_{i},r_{j})) = 0, $$

where the equilibrium strategies $x_{i}^{*}(r_{i},r_{j}),x_{j}^{*}(r_{i},r_{j})$ are given by Lemma 1. After setting *r*_*i*_ = *r*_*j*_ = *r*, the first-order condition for fitness maximization (8) can be written

9 $$ \frac{a^{2} b^{2} (-1 \!+ t^{2}) \left( b (1 \!+ t) (1 + r^{2} + 2 r t) + 2 (1 + c) \left( t + r (1 + t + r t^{2})\right)\right)}{\left( b (-1 \!+ r) (1 \!+ t) - 2 (1 \!+ c) (-1 \!+ r t)\right) \left( b (1 + r) (1 + t) + 2 (1 + c) (1 + r t)\right)^{3}} = 0 $$

from which the candidate for the symmetric ES conjecture can be solved. We note that, because there is a factor (−1 + *t* ^2^) in the numerator, the first-order condition (9) vanishes for *t* = −1 and *t* = 1 and the ES conjecture is not defined for these limit values.

#### **Proposition 2**

*In Cournot competition with ORPs, the evolutionarily stable conjecture is*

10 $$ \begin{array}{lllllll} &r^{E} = -\frac{(1+c+bt)(-1+t^{2})+ \sqrt{A}}{(-1+t)\left( b+bt+2(1+c)t^{2}\right)}, \\ &where\quad A=(-1+t)^{3}\left( b^{2}(1+t)^{3}-(1+c)^{2}\left( 1+t(3+4t)\right)\right). \end{array} $$

#### *Remark 3*

The conjecture *r* ^*E*^(*t*) assumes generally negative values but becomes positive for a range of negative t and approaches unity as *t* →−1. This implies that, for sufficiently spiteful ORPs, the reaction functions slope up and the quantities decrease below those in the CNE (see Remark 1).

#### **Corollary 1**

*The ES conjecture* *r* ^*E*^(*t*)*is strictly decreasing in* t *in the Cournot game.*

#### *Remark 4*

Corollary 1 implies that *r* ^*E*^(*t*) crosses *r* ^∗^ only once and this is exactly at *t* = 0. This can be seen by observing that *r* ^*E*^(0) = *r* ^∗^. This also implies that the point *t* = 0 where ORPs change between spiteful and altruistic also determines whether the equilibrium quantities with the ES conjectures are lower or higher than those in the CCE. With self-regarding preferences *t* = 0 the quantities (4) are equal as well.

**Fig. 1:**
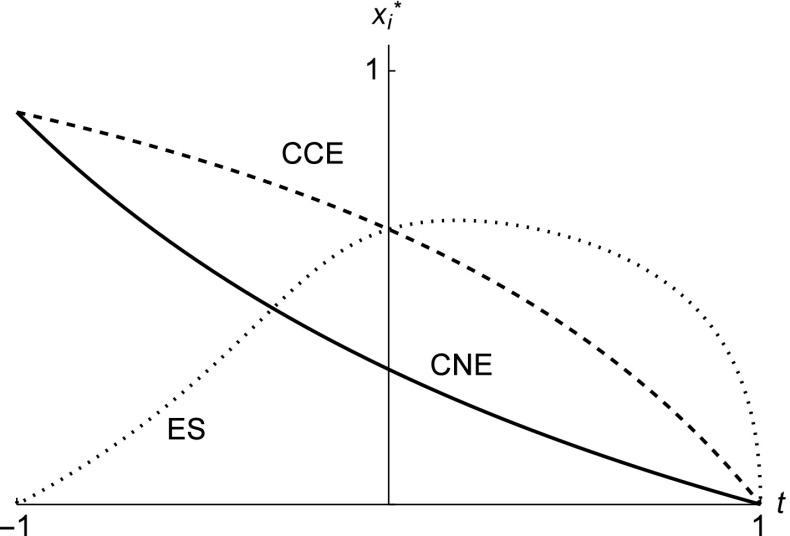
Equilibrium quantity as function of *t* in the Cournot duopoly for the Nash conjectures (solid line), the consistent conjectures (dashed line), and the ES conjectures (dotted line). The parameter values are *a* = 2, *b* = 0.95, and *c* = 0.05

Figure 1 illustrates the situations of Remarks 3 and 4 and depicts the equilibrium quantities as function of *t*. The CCE and the ES quantities intersect at *t* = 0. The ES quantity decreases as the ORPs get increasingly spiteful while the CCE and CNE quantities increase.

## The Bertrand duopoly game

### The model

As in Cournot competition, the players make simultaneous decisions in a one-shot game. Now the strategies $x_{i} \in \mathbb {R}^{+}\cup \{0\}$ are prices. The payoff functions are

11 $$ g_{i}(x_{i},x_{j}) = x_{i}\left( \frac{a(1-b)-x_{i}+bx_{j}}{1-b^{2}}\right)-\frac{c}{2}\left( \frac{a(1-b)-x_{i}+bx_{j}}{1-b^{2}}\right)^{2} $$

and they are formed similarly as in the Cournot case but from the demand function that gives quantity as function of prices.^2^ Firms maximize utility given by Eq. 2 where we replace the Cournot payoff functions by the Bertrand payoff functions (11). The payoff and utility functions are smooth also in the Bertrand case. The reaction functions are solved from the first-order condition *G*_*i*_(*x*_*i*_,*x*_*j*_;*r*_*i*_) = 0 whose left-hand side is given by

12 $$\begin{array}{@{}rcl@{}} G_{i}(x_{i},x_{j};r_{i}) &:=& \frac{\partial u_{i}}{\partial x_{i}}(x_{i},x_{j}) + r_{i}\frac{\partial u_{i}}{\partial x_{j}}(x_{i},x_{j}) \\ &=& -\frac{1}{(-1+b^{2})^{2}} \left( -a (-1 + b) \left( b c (r_{i} + t) + b^{2} (1 + r_{i} t) - (1 + c) (1 + r_{i} t)\right) \right.\\ &&+ 2 x_{i} + c x_{i} + (2 + c) r_{i} t x_{j} + b^{3} (1 + t) (r_{i} x_{i} + x_{j}) - b (1 + c) (1 + t) \\ &&\left.\cdot (r_{i} x_{i} + x_{j}) + b^{2} \left( (-2 + c t) x_{i} + r_{i} (c - 2 t) x_{j}\right)\right). \end{array} $$

As in the Cournot case, the Bertrand payoff functions are quadratic and the reaction functions are linear. For the special case of zero conjectures, *r*_*i*_,*r*_*j*_ = 0, the corresponding equilibrium is again called the Cournot-Nash equilibrium.

#### **Lemma 2**

*The equilibrium prices as function of arbitrary conjectures* $x_{i}^{*}(r_{i},r_{j})$ *exist for all feasible* *a*, *b*, *c* *and* t *and are given by*

13 $$ \begin{array}{lllllll} x_{i}^{*}(r_{i},r_{j}) = &\left( a \left( (-1 + b^{2} - c + b c r_{i}) (2 + c + b^{2} (-1 + r_{j}) - b (1 + r_{j} + c r_{j}))\right. \right.\\ &+ (-1 + b)^{2} b (1 + b) (-1 + r_{i} r_{j}) t + \left( b^{2} (-1 + r_{i}) - (2 + c) r_{i} \right.\\ &\left.\left.\left. + b (1 + c + r_{i})\right) \left( b c + b^{2} r_{j} - (1 + c) r_{j}\right) t^{2}\right)\right) / \left( b^{4} (-1 + r_{i} r_{j})\right. \\&\cdot (1 + t)^{2} + b^{3} (2 + c) (r_{i} + r_{j}) (-1 + t^{2}) - b (1 + c) (2 + c) (r_{i} + r_{j}) \\&\cdot (-1 \!+ t^{2}) \,+\, (2 \!+ c)^{2} (\!-1 \!+ r_{i} r_{j} t^{2}) \!+ b^{2} \left( 5 \!- r_{i} r_{j} \!- c \left( \!-2 \!+ (2 \!+ c) r_{i} r_{j}\right)\right. \\&\left.\left. + 2 t - 2 r_{i} r_{j} t + \left( (1 + c)^{2} - (5 + 2 c) r_{i} r_{j}\right) t^{2}\right)\right). \end{array} $$

We are again only interested in conjectures that are continuous in the parameters *a*, *b*, *c* and *t*. Also, as the game is symmetric, we can assume that the conjectures are symmetric as well, *r*_*i*_ = *r*_*j*_ = *r*.

#### *Remark 5*

By inspecting the derivative of the reaction function $x_{i}^{*}(x_{j};r)$ w.r.t *x*_*j*_ we can obtain information about its slope for different ranges of t and r. For zero ORPs *t* = 0 the slope is (*b*(1 + *c* − *b*(*b* + *c* *r*)))/ (2 − 2*b* ^2^ + *c* + *b* ^3^ *r* − *b*(1 + *c*)*r*) > 0 and the reaction function is upward-sloping for any *r* ∈ [−1,1] in the Bertrand duopoly game. For *t* ≠ 0 the slope is

$$-\frac{b^{2} r (c - 2 t) + (2 + c) r t + b^{3} (1 + t) - b (1 + c) (1 + t)}{2 + c + b^{3} r (1 + t) - b (1 + c) r (1 + t) + b^{2} (-2 + c t)} $$

and the reaction function is downward-sloping when

$$\frac{2 b (1 - b^{2} + c)}{2 + b^{2} (-2 + c) + c}<r<1, \quad \frac{b \left( 1 + c - b (b + c r)\right)}{b^{3} - b (1 + c) - 2 b^{2} r + (2 + c) r}<t<1 $$

or when

$$-1<r<0, \quad -1<t<\frac{b \left( 1 + c - b (b + c r)\right)}{b^{3} - b (1 + c) - 2 b^{2} r + (2 + c) r}. $$

The upward-sloping reaction function (with strategic complements) is now the standard case except for certain ranges of positive conjectures and altruistic preferences or for negative conjectures and spiteful preferences, in which cases the reaction function can slope downwards, thus yielding prices as strategic substitutes. See also Remark 1.

### The consistent conjectures

In the Bertrand duopoly game, the equation that determines the consistent conjectures, i.e. Eq. 5 with functions (12), is

14 $$ r = -\frac{b^{2} r (c - 2 t) + (2 + c) r t + b^{3} (1 + t) - b (1 + c) (1 + t)}{2 + c + b^{3} r (1 + t) - b (1 + c) r (1 + t) + b^{2} (-2 + c t)}. $$

#### **Proposition 3**

*In Bertrand competition with ORPs the consistent conjecture is*

15 $$ r^{*} = \frac{-\left( 2-2b^{2}+(1+b^{2})c\right) + (1 - b^{2})\sqrt{-4 b^{2} + (2 + c)^{2}}}{2 b (-1 + b^{2} - c)}. $$

#### *Remark 6*

Proposition 3 shows that (as in Remark 2) assuming ORPs does not change the consistent conjectures in the Bertrand duopoly game.

### Evolutionarily stable conjectures

As in the Cournot case, we use the equilibrium prices with arbitrary conjectures $\left (x_{i}^{*}(r_{i},r_{j}),x_{j}^{*}(r_{i},r_{j})\right )$ given by Lemma 1. Player *i*’s first-order condition for fitness maximization is of the form

16 $$ \frac{\partial}{\partial r_{i}} g_{i} \left( x_{i}^{*}(r_{i},r_{j}),x_{j}^{*}(r_{i},r_{j})\right) = 0. $$

After setting *r*_*i*_ = *r*_*j*_ = *r* we get the first-order condition for fitness maximization into a form from which the symmetric conjecture *r* can be solved:

17 $$ \begin{array}{lllllll} &\left( a^{2} b^{2} (\!-1 \,+\, b^{2}) (-1 \,+\, t^{2}) \left( b^{3} (1 \,+\, t) (1 \!+ r^{2} \!+ 2 r t) - b (1 \!+ c) (1 + t) (1 + r^{2} + 2 r t) \right.\right.\\ &\left.\left.\!+ b^{2} \left( r^{2} (c \,-\, 2 t) t \!+ (-2 \,+\, c) r (1 \,+\, t) \,+\, t (-2 \,+\, c t)\right) \!+ (2 + c) \left( t + r (1 + t + r t^{2})\right)\right)\right) \\ / &\left( \left( 2 + c - b (1 + r + c r) - (2 + c) r t + b (1 + c + r) t + b^{2} (-1 + r) (1 + t)\right) \right.\\ &\left.\cdot \left( b^{2} (1 + r) (1 + t) + b \left( -1 + r (1 + c - t) + t + c t\right) - (2 + c) (1 + r t)\right)^{3}\right) = 0. \end{array} $$

We note that the ES conjectures are not defined for the limit values of *t* = −1 and *t* = 1. This is because the factor (−1 + *t* ^2^) in the numerator of the first-order condition for fitness maximization (17) makes it vanish for these values of *t*.

#### **Proposition 4**

*In Bertrand competition with ORPs the ES conjecture is given by*

$$r^{E}= \left\{\begin{array}{lllllll} r^{E,1} & \quad \text{for} \quad t \in (-1,1)\setminus \{t^{-}\}\cup\{t^{+}\}\\ r^{E,2} & \quad \text{for} \quad t \in \{t^{-}\}\cup\{t^{+}\} \end{array}\right. $$

suchthat

$$\begin{array}{lllllll} r^{E,1} \,=\,& \left( \!-4 b^{2} (-1 \,+\, t) (1 \,+\, t)^{2} \,-\, b^{4} (\!-2 \,+\, c) (\!-1 \,+\, t) (1 \,+\, t)^{2} \,+\, (2 + c) (-1 + t) (1 + t)^{2} \right.\\ & - 2 b^{5} (-1 \!+ t) t (1 \!+ t)^{2} - 2 b (1 \!+ c) (-1 \!+ t) t (1 + t)^{2} + 2 b^{3} (2 + c) (-1 + t) \\&\left.\cdot t (1 \,+\, t)^{2} \,+\, \sqrt{A} \right) / \left( 2 (\!-1 \,+\, b^{2}) (-1 \,+\, t^{2}) \left( b^{2} (c \,-\, 2 t) t \!+ \!(2 + c) t^{2} + b^{3} (1 + t) \right.\right.\\ & \left.\left.- b (1 + c) (1 + t)\right)\right) \end{array} $$

where

$$A = (-1 + b^{2})^{4} (1- t)^{3} (1 + t)^{2} \left( -4 b^{2} (1 + t)^{3} + (2 + c)^{2} \left( 1 + t (3 + 4 t)\right)\right) $$

and

$$r^{E,2} = \frac{ -(2 + c) t - b^{3} (1 + t) + b (1 + c) (1 + t) + b^{2} t (2 - c t)}{(1 + t) \left( 2 + b^{2} (-2 + c) + c + 2 b^{3} t - 2 b (1 + c) t\right)} $$

and

$$t^{\mp} = \frac{b - b^{3} \,+\, b c - b^{2} c \mp \sqrt{4 (2- 2 b^{2} \,+\, c) (b - b^{3} \,+\, b c) \,+\, (-b + b^{3} - b c + b^{2} c)^{2}}}{2 (2 - 2 b^{2} + c)}. $$

#### *Remark 7*

As in the Cournot case, the evolutionarily stable conjecture in the Bertrand game coincides with the consistent conjecture exactly at *t* = 0. The evolutionarily stable conjecture is generally positive but obtains negative values as t approaches unity.

#### **Corollary 2**

*The ES conjecture* *r* ^*E*^(*t*)*is strictly decreasing in* t *in the Bertrand game.*

**Fig. 2:**
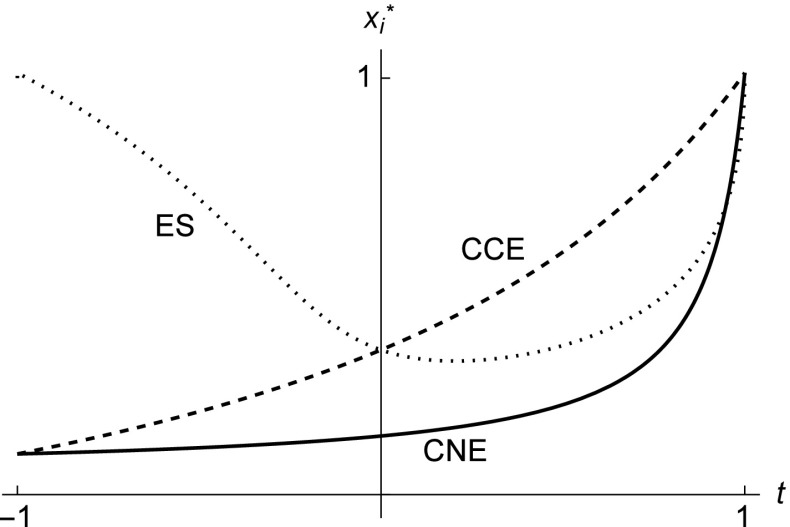
Equilibrium price as function of *t* in the Bertrand duopoly for the Nash conjectures (solid line), the consistent conjectures (dashed line), and the ES conjectures (dotted line). The parameter values are *a* = 2, *b* = 0.95, and *c* = 0.05

Figure 2 illustrates the behavior of the equilibrium prices in the Bertrand duopoly game for different *t*. The CCE and ES prices intersect at *t* = 0. As we move to the left towards increasingly spiteful preferences, the equilibrium price with the ES conjecture increases while the CCE and CNE prices decrease. As an increasing price indicates increasing cooperative behavior, this result is qualitatively the same as in the Cournot duopoly game (see Fig. 1).

## Discussion

Holt (1985) argues that the concept of CCE can be refuted on the basis of laboratory evidence. In the Cournot duopoly game the CCE predicts higher quantities than the CNE even in the presence of ORPs (see Fig. 1), but laboratory experiments show that quantities are usually equal to or lower than those in the CNE. For example, Huck et al. (2001) find that average quantities in one-shot Cournot duopoly markets roughly correspond to the CNE quantities, and Suetens and Potters (2007) survey Bertrand experiments and find that tacit collusion is more frequent with price than with quantity. Our results suggest that a variety of behavioral outcomes can be supported by assuming conjectures and/or other-regarding preferences in both the Cournot and the Bertrand duopoly games. While cooperative behavior can generally be explained by altruistic preferences, our novel result is that spiteful preferences also explain cooperative outcomes if we assume that the players have evolutionarily stable conjectures. Figure 1 implies that spiteful Cournot players with ES conjectures can actually choose quantities that are close to the CNE quantities or even close to the collusive quantities. On the other hand, altruistic players with ES conjectures would end up playing quantities that are higher than even the CCE quantities. Similar arguments hold for the Bertrand duopoly game as Fig. 2 demonstrates. These results shed new insights also to explaining the laboratory evidence of Huck et al. (2001) and others.

Burnham (2014) presents the concepts of proximate and ultimate mechanisms from evolutionary biology to economic theory. Ultimate mechanisms determine what kind of behavior survives in evolution in the long run. Proximate mechanisms explain what motivates behavior in the short run. In this view, the consistent conjectures are the products of proximate mechanisms and the ES conjectures arise from ultimate mechanisms. Whereas prior research has shown that the ultimate and proximate mechanisms produce the same conjectures if the duopolists are self-regarding (Müller and Normann 2005; Possajennikov 2009), we show that the ES conjectures are different from the consistent conjectures if we allow a simple type of other regarding behavior.

It should be noted that our models of Cournot and Bertrand competition are specific in terms of linear demand functions and quadratic costs. Also, the utility function model is restrictive because only a very simple linear utility function is used and the ORP parameter is assumed to be symmetric among the firms. These restrictions limit the generalizations that can be drawn from the results.

Unlike Bester and Güth (1998) and others who have modeled the evolution of altruism and spite in the linear-quadratic context, we leave the ORP parameter untouched by evolutionary forces. This is in part a modeling decision: If both the ORP parameter and the conjecture were subject to selection pressure, we would lose analytical tractability. However, another rationale is that it allows us to explain situations where selection pressure focuses on nonzero conjectures but where there are degrees of freedom in terms of distortions in payoff maximization. Therefore, the conjecture is the expression of the tendency that evolves (Trivers 1971) whereas the ORP parameter allows, for example, the characterization of phenotypic variability. Our results then suggest that in two of the most studied duopoly games with linear-quadratic payoffs, the Cournot and the Bertrand games, this variability in the ES conjectures is nonzero.

The assumption of the common ORP parameter *t* for both players can be justified on several grounds. Here, too, the assumption maintains analytic tractability. However, one can also quickly imagine situations where exogenous factors demand that assumption, such as when the individual utilities are common knowledge, or when firms explicitly agree on a common *t* parameter (as in colluding). Even in a population of dissimilar phenotypes, evolutionary forces may favor interactions between phenotypically similar individuals (Antal et al. 2009).

Müller and Normann (2005) argue that because the consistent conjecture is equal to the ES conjecture, evolutionary stability can rationalize consistency. Possajennikov (2009) points out that the ES conjecture is consistent because the players with consistent conjectures correctly anticipate the other player’s reaction function and therefore solve the correct first-order conditions. Our results challenge both of these arguments. This is because in the presence of ORPs (i) evolutionary stability cannot rationalize consistency, and (ii) players with consistent conjectures do not solve the correct first-order conditions.

Future research should extend our analysis by allowing the model of ORPs be more general or by allowing distortions in the symmetric ORP parameters. Also, the results could be extended to cover other games such as public goods games. Yet another extension would be an evolutionary model that allows the evolution of both ORPs and conjectures. The players could, for example, first develop evolutionarily stable conjectures that depend on the ORP parameters and then subject the ORPs to evolutionary pressure.

## Proofs

### Proof of lemma 1

Solve the equilibrium quantities $x_{i}^{*},x_{j}^{*}$ from the pair of first-order conditions the left-hand sides of which are given by Eq. 3. Writing the derivative of *F*_*i*_(*x*_*i*_,*x*_*j*_;*r*_*i*_) as

$$\frac{\partial}{\partial x_{i}} F_{i}(x_{i},x_{j};r_{i}) = -2 - 2 c - b r_{i} (1 + t) $$

we see that the second-order condition is satisfied. The candidate is Eq. 4, which has a denominator and a numerator that are quadratic polynomials in each of the parameters. We note that all the other parameters in the denominator are restricted except *c*, which can increase without limit. It is therefore instructive to write the denominator as a polynomial function of *c* and analyze the ranges of its coefficients. We see from the following form of the denominator

$$\begin{array}{lllllll} & \underbrace{4 - 4 r_{i} r_{j} t^{2} + b^{2} (-1 + r_{i} r_{j}) (1 + t)^{2} - 2 b (r_{i} + r_{j}) (-1 + t^{2})}_{\text{Range: }(0,8)}\\ +& \underbrace{\left( 8 - 8 r_{i} r_{j} t^{2} - 2 b (r_{i} + r_{j}) (-1 + t^{2})\right)}_{\text{Range: }(0,16)} \cdot c \\ +&\underbrace{\left( 4 - 4 r_{i} r_{j} t^{2}\right)}_{\text{Range: }(0,8)} \cdot c^{2} \end{array} $$

that as the coefficients of *c* have strictly positive ranges and *c* ≥ 0, the denominator cannot assume nonpositive values. The limit values of the denominator are − 4(1 + *c*)^2^(−1 + *r*_*i*_ *r*_*j*_) as *t* →−1 and 4(*b* ^2^ − (1 + *c*)^2^)(−1 + *r*_*i*_ *r*_*j*_) as *t* → 1, which are also both nonnegative.

### Proof of proposition 1

From Eq. 6, we get two candidates for the consistent conjectures,

$$r^{*\pm} = \frac{-(1+c)\pm\sqrt{-b^{2}+(1+c)^{2}}}{b}. $$

The numerator of the positive root equals zero when *b* = 0 and $-(1 + c) + \sqrt {c (2 + c)} \in (-1,0)$ when *b* = 1. Because $\sqrt {-b^{2}+(1+c)^{2}}$ is decreasing in *b*, for intermediate values of *b* the numerator of the positive root is strictly in (−1,0). The numerator of the negative root, however, equals − 2(1 + *c*) < 0 when *b* = 0 and $-(1 + c) - \sqrt {c (2 + c)} \in (-\infty ,-1]$ when *b* = 1. From this we already see that the negative root cannot always be in [−1,1]. Furthermore, when *c* ≫ *b*, the term with *b* in the numerator of the positive root loses its impact on its value and the terms with *c* cancel each other. Therefore only the positive root *r* ^∗+^ is well-behaved and in the closed interval [−1,1] for all feasible *b* and *c* but the negative root *r* ^∗−^ is not. Because *b* < 1, the positive root *r* ^∗+^ is also strictly below zero for all feasible values of *b*, *c*.

### Proof of proposition 2

At Step 1 we determine the ES conjecture candidate from the first-order condition for fitness maximization (9) and show that the candidate satisfies the limit values. At Step 2 we show that the candidate is the best response against itself and that the population of players with *r* ^*E*^’s survives a mutant invasion, i.e. the candidate is an ES strategy (Maynard Smith 1982).

#### Step 1

Solve (9) for *r* to get two candidates for the ES conjecture,

$$r^{E\pm} = -\frac{(1+c+bt)(-1+t^{2})\pm \sqrt{A}}{(-1+t)\left( b+bt+2(1+c)t^{2}\right)}, $$

where *A* is as given in Eq. 10. We make the following observations from the candidates:

- First we note that the denominators of the candidates *r* ^*E*±^ are always nonzero. Furthermore, the term *A* is a product of two negative factors and therefore positive. Thus the candidates *r* ^*E*±^ are continuous for all feasible *b*, *c* and *t*.
- The positive root *r* ^*E*+^ ∈ [−1,1]. This is shown by first examining limit behavior and then behavior between the limits. The positive root approaches 1 when *t* →−1 and − 1 when *t* → 1. We thus have to show that *r* ^*E*+^ = −1 only at *t* = −1 and *r* ^*E*+^ = 1 only at *t* = 1. Rearranging the equation *r* ^*E*+^ − 1 = 0 such that the square-root term is at the right-hand side and all the other terms at the left-hand side, squaring each side and then moving all terms back to the left-hand side results in an equation
  $$2 (1 + b + c) (-1 + t^{2})^{2} \left( b + b t + 2 (1 + c) t^{2}\right) = 0 $$
  that holds only when *t* = −1 or *t* = 1. However, knowing that *r* ^*E*+^ = −1 at *t* = 1, we can conclude that *t* = −1 is the unique solution of the equation *r* ^*E*+^ − 1 = 0. Using a similar procedure for the equation *r* ^*E*+^ + 1 = 0 yields the conclusion that this equation holds exactly at *t* = 1.
- For the negative root, there are values of *b*, *c* and *t* that yield the candidate out of its limits. For example, when *b* = 1/2, *c* = 0 and *t* = 1/3 we have *r* ^*E*−^ = −3. This is enough to show that *r* ^*E*−^∉[−1,1] for some values of *b*, *c* and *t*.

Therefore, we accept only the positive root *r* ^*E*^ = *r* ^*E*+^. This concludes Step 1.

#### Step 2

If the expression

18 $$ f_{i}(r^{E},r^{E}) \geq f_{i}(r,r^{E}), $$

where *r* ∈ [−1,1] is an arbitrary conjecture, holds strictly for *r*≠*r* ^*E*^, then *r* ^*E*^ is the unique best reply against itself (Müller and Normann 2005). We can inspect the function *f*_*i*_(*r*, *r* ^*E*^) and show that *r* ^*E*^ is its unique maximizer on [−1,1]. The function *f*_*i*_(*r*, *r* ^*E*^) is a rational function of the form *P*(*r*)/*Q*(*r*) where *P*(*r*) and *Q*(*r*) are second-degree polynomials in *r*. Taking its derivative in *r* leads to a form (*P* ^′^(*r*)*Q*(*r*) − *P*(*r*)*Q* ^′^(*r*))/*Q*(*r*)^2^, which, after canceling common factors, has a first-degree polynomial in *r* at the numerator and a third-degree polynomial at the denominator. Therefore, there is just one maximizer to *f*_*i*_(*r*, *r* ^*E*^) and by substitution it can be shown to be *r* ^*E*^. The complicated expressions of *P*(*r*) and *Q*(*r*) are too long to be presented here, but are available from the Author upon request.

### Proof of corollary 1

Step 1 in the Proof of Proposition 2 shows that the limit values of *r* ^*E*^(*t*) for *t* = −1 and *t* = 1 are 1 and − 1, respectively. Therefore, to show that *r* ^*E*^(*t*) is strictly decreasing in *t* ∈ (−1,1), we must show that *r* ^*E*^(*t*) crosses an arbitrary constant conjecture *r* ∈ (−1,1) (i.e. a horizontal line in (*t*, *r*)-plane) only once in *t* ∈ (−1,1). We form the equation *r* ^*E*^(*t*) − *r* = 0 and manipulate it such that we move square-root terms on the right-hand side, square each side and move everything back to the left-hand side. A simplified equation

19 $$ \frac{b (1 + t) (1 + r^{2} + 2 r t) + 2 (1 + c) \left( t + r (1 + t + r t^{2})\right)}{b + b t + 2 (1 + c) t^{2}}=0 $$

follows. There is an upwards-opening quadratic polynomial in *t* in the numerator of the left-hand side of Eq. 19 that has at most two (real) roots. Because *r* ^*E*^(*t*) − *r* is smooth in *t*, we know that at least one root must always be in (−1,1). Therefore, if the other root is outside of (−1,1), then the quadratic has a different sign at the point *t* = −1 than at the point *t* = 1. Indeed, we find that the quadratic is

$$\begin{array}{lllllll} &2 (1 + c) (-1 + r^{2}) \quad \text{for} \quad t=-1,\\ &2 (1 + b + c) (1 + r)^{2} \quad \text{for} \quad t=1. \end{array} $$

These have different signs for all *r* ∈ (−1,1) and for all feasible *b*, *c*, proving that there is only one *r* that satisfies (19) in (−1,1).

### Proof of lemma 2

Solve the equilibrium prices $x_{i}^{*},x_{j}^{*}$ from the pair of first-order conditions the left-hand sides of which are given by Eq. 12. Writing the derivative of *G*_*i*_(*x*_*i*_,*x*_*j*_;*r*_*i*_) as

$$\frac{\partial}{\partial x_{i}} G_{i}(x_{i},x_{j};r_{i}) = \frac{-2 - c - b^{3} r_{i} (1 + t) + b (1 + c) r_{i} (1 + t) + b^{2} (2 - c t)}{(-1 + b^{2})^{2}} $$

we see that the second-order condition is satisfied. Both the denominator and numerator of Eq. 13 are quadratic polynomials in *t*. The equilibrium prices thus exist if the denominator has no zeros in − 1 < *t* < 1. As in the Cournot case (see Proof of Lemma 1), we examine the zeros by writing the denominator as a function of *c*. We can express the denominator in the following form

$$\begin{array}{lllllll} &\underbrace{(-1 + b^{2}) \left( 4 - 4 r_{i} r_{j} t^{2} + b^{2} (-1 + r_{i} r_{j}) (1 + t)^{2} + 2 b (r_{i} + r_{j}) (-1 + t^{2})\right)}_{\text{Range: } (-8,0)} \\ + & \underbrace{\left( -4 + 4 r_{i} r_{j} t^{2} - 3 b (r_{i} + r_{j}) (-1 + t^{2}) + b^{3} (r_{i} + r_{j}) (-1 + t^{2}) - 2 b^{2} (-1 + r_{i} r_{j}) (1 + t^{2})\right)}_{\text{Range: }(-8,0)} \cdot c \\ + & \underbrace{\left( -1 + b (r_{i} + r_{j} - b r_{i} r_{j}) + (b - r_{i}) (b - r_{j}) t^{2}\right)}_{\text{Range: }(-4,0)} \cdot c^{2} \end{array} $$

and see that all the coefficients of *c* are strictly negative and thus the denominator cannot assume nonnegative values. The limit values of the denominator are − (−1 + *b* ^2^)(2 + *c*)^2^(−1 + *r*_*i*_ *r*_*j*_) as *t* →−1 and (−1 + *b* ^2^)(4*b* ^2^ − (2 + *c*)^2^)(−1 + *r*_*i*_ *r*_*j*_) as *t* → 1 and these limits are strictly negative.

### Proof of proposition 3

Solving Eq. 14 gives two candidates for the consistent conjectures:

$$r^{*\pm} = \frac{-\left( 2-2b^{2}+(1+b^{2})c\right)\pm (1 - b^{2})\sqrt{-4 b^{2} + (2 + c)^{2}}}{2 b (-1 + b^{2} - c)}. $$

The denominator of the candidates is strictly negative for all feasible *b* and *c*; furthermore, *r* ^∗+^ < *r* ^∗−^. To show that the positive root is the unique consistent conjecture within set [−1,1], Eq. 14 can be simplified to assume the form (*b* ^3^ − *b*(1 + *c*))*r* ^2^ + (2 + *b* ^2^(−2 + *c*) + *c*)*r* + *b* ^3^ − *b*(1 + *c*) = 0, the left hand side of which is a quadratic polynomial in *r* that has zeros *r* ^∗+^ and *r* ^∗−^. We can see that, for all feasible values of *b* and *c*, this polynomial is negative when *r* = 0, positive when *r* = 1, and negative when *r* →*∞*. Because *r* ^∗+^ < *r* ^∗−^ we then know that the positive root *r* ^∗+^ is in the set [−1,1] for all feasible *b* and *c*, while the negative root is not in the set [−1,1].

### Proof of proposition 4

This proof is similar to the Proof of Proposition 2.

#### Step 1

The first-order condition (17) is of the form *P*(*r*)/*Q*(*r*) and the solution *r* ^*E*,1^ is obtained from *P*(*r*) = 0. *P*(*r*) is a second-degree polynomial in *r*. The denominator of the solution to *P*(*r*) = *A*(*t*)*r* ^2^ + *B*(*t*)*r* + *C*(*t*) = 0 is zero for some *t*, namely, when *A*(*t*) = 0. These zeros are given by *t* ^−^ and *t* ^+^. For these zeros in the denominator we consider the reduced form *P*(*r*) = *B*(*t*)*r* + *C*(*t*) = 0, which has *r* ^*E*,2^ as the unique solution.

The corresponding negative root to *r* ^*E*,1^ is not within [−1,1] for all *b*, *c* and *t*. For example, for *b* = 1/3, *c* = 0 and *t* = 1/5 the negative root equals 7. The positive root *r* ^*E*,1^ ∈ [−1,1] for *t* ∈ (−1,1) ∖{*t* ^−^}∪{*t* ^+^}, and this is seen by examining it similarly as in the Proof of Proposition 2. First we note that $\lim _{t \to -1}r^{E,1}=1$ and $\lim _{t \to 1}r^{E,1}=-1$. Then we show that *r* ^*E*,1^ = 1 only at *t* = −1, i.e. we rearrange the equation *r* ^*E*,1^ − 1 = 0 such that the square-root term is on the right-hand side, square each side, and then move all back to the left-hand side. This results in an equation

$$\frac{(-1 + b)^{2} (2 + 2 b + c) (1 + t)^{2}}{b^{2} (c - 2 t) t + (2 + c) t^{2} + b^{3} (1 + t) - b (1 + c) (1 + t)}=0, $$

which holds only for *t* = −1. A similar procedure can be followed to obtain the limit for *t* = 1.

#### Step 2

As in the Proof of Proposition 2, we inspect the inequality

20 $$ g_{i}(r^{E},r^{E}) \geq g_{i}(r,r^{E}) $$

where *r* ∈ [−1,1] is an arbitrary conjecture. However, this time we inspect separately the conjectures *r* ^*E*,1^ and *r* ^*E*,2^.

When *t* ∈ (−1,1) ∖{*t* ^−^}∪{*t* ^+^}, the function *g*_*i*_(*r*, *r* ^*E*,1^) is a rational function of the form *P*(*r*)/*Q*(*r*) where *P*(*r*) and *Q*(*r*) are second-degree polynomials in *r*. Taking its derivative in *r* leads again to a form (*P* ^′^(*r*)*Q*(*r*) − *P*(*r*)*Q* ^′^(*r*))/*Q*(*r*)^2^, which simplifies to a rational function that has a first-degree polynomial in *r* at the numerator and third-degree polynomial at the denominator. Therefore, there is a unique maximizer to *g*_*i*_(*r*, *r* ^*E*,1^) and by substitution it can be shown to be *r* ^*E*,1^. The complicated expressions of *P*(*r*) and *Q*(*r*) are too long to be presented here, but are available from the Author upon request.

When *t* ∈{*t* ^−^}∪{*t* ^+^}, the function *g*_*i*_(*r*, *r* ^*E*,2^) is again of the form *P*(*r*)/*Q*(*r*) with second-degree polynomials in *r*. The same arguments hold as above, i.e. we get the result that *r* = *r* ^*E*,2^ is the unique maximizer of *g*_*i*_(*r*, *r* ^*E*,2^) in [−1,1].

### Proof of corollary 2

This proof is similar to the Proof of Corollary 1. The limit values of the ES conjecture *r* ^*E*^(*t*) are $\lim _{t\to -1}r^{E}(t)=1$ and $\lim _{t\to 1}r^{E}(t)=-1$. Therefore, it remains to show that *r* ^*E*,1^(*t*) − *r* = 0 has only one root at *t* ∈ (−1,1) for all arbitrary constant conjectures *r*. Manipulating the equation such that we move square-root terms on the right-hand side, square each side and move everything back to the left-hand side results in a simplified equation that has a numerator of the form

21 $$ \begin{array}{lllllll} &b^{3} (1 + t) (1 + r^{2} + 2 r t) - b (1 + c) (1 + t) (1 + r^{2} + 2 r t) + b^{2} \left( r^{2} (c - 2 t) t \right.\\ &\left.+ (-2 + c) r (1 + t) + t (-2 + c t)\right) + (2 + c) \left( t + r (1 + t + r t^{2})\right). \end{array} $$

The expression (21) is a downwards-opening quadratic polynomial in *t*. Knowing that at least one of the roots is in (−1,1) it remains to show that (21) has a different sign on *t* = −1 than on *t* = 1 for all *r* ∈ (−1,1). We find that the expression reduces to

$$\begin{array}{lllllll} &-(-1 + b^{2}) (2 + c) (-1 + r^{2}) \quad \text{for} \quad t=-1,\\ &(-1 + b)^{2} (2 + 2 b + c) (1 + r)^{2} \quad \text{for} \quad t=1. \end{array} $$

These have different signs for all *r* ∈ (−1,1) and for all feasible *b*, *c*. Therefore, Eq. 21 is equal to zero only for one value of *r*.

## Footnotes

1 The results in this section and in Section 3 can be extended for finite populations by using the finite population ESS (Schaffer 1988). See also Müller and Normann (2007).

2 To simplify the expressions we use *c*/2 as the cost function parameter instead of *c* as in the Cournot case. This does not change the interpretations of the results in any way.
